# Women’s awareness, knowledge, attitudes, and behaviours towards nutrition and health in Pakistan: Evaluation of kitchen gardens nutrition program

**DOI:** 10.1371/journal.pone.0291245

**Published:** 2023-09-14

**Authors:** Nadia Shah, Sidra Zaheer, Nilofer Fatimi Safdar, Tahir Turk, Shahkamal Hashmi

**Affiliations:** 1 School of Public Health, Dow University of Health Sciences, Karachi, Pakistan; 2 Communication Partners International (CPI), Springfield, NSW, Australia; Xiangtan University, CHINA

## Abstract

**Introduction:**

Vulnerability to malnutrition is very high with low-income women and their children in rural Balochistan with contributing factors including lack of awareness about proper nutrition, low literacy, scarcity of vegetables and fruit, and low purchasing power of households. The Food and Agriculture Organization’s kitchen garden program provides resources to improve nutrition and health knowledge and promote healthy eating practices. The objective of this study was to assess nutrition and health awareness, knowledge, attitudes, behavioural intentions/behaviours (AKAB) of women who attended the kitchen garden program and trainings.

**Materials and methods:**

A community based cross-sectional survey (N = 209) using a two-stage cluster sampling method was used to select households with survey participants being mothers with children under five years of age. A pretested questionnaire was administered via face-to-face surveys by trained enumerators in two districts of Balochistan province of Pakistan. Nutrition and health AKAB were constructed indices. Chi-square tests compared statistical differences in AKAB by women attending against a control group who did not-attend kitchen garden interventions. Binary logistic regression analyses were performed to assess kitchen garden program outcomes against key AKAB indicators, while adjusting for covariates.

**Results:**

Significant differences (p<0.001) were identified between intervention and control groups with women attending kitchen garden being more aware of the components of kitchen garden (65.8% vs 36.8%), and more knowledgeable about causes of illnesses caused by poor nutrition including, iron deficiency anemia, pregnancy, and unborn child health complications, compared to women not attending kitchen gardens program. Logistic regression analysis identified women attending kitchen gardens also had higher odds of being more knowledgeable (OR = 1.59, 95%CI 1.27–1.99, p<0.001), having improved attitudes (OR = 4.86, 95%CI 2.77–8.53, p <0.001), and behavioural intentions/behaviours (OR = 1.98, 95%CI 1.26–3.12, p = 0.003) towards improved nutrition and health.

**Conclusions:**

Substantial opportunities exist for achieving improved nutrition and health outcomes with vulnerable groups in Balochistan, through greater participation in kitchen gardens behavioural change programs and interventions. As part of scaling-up efforts, academically rigorous project evaluations should be institutionalized for continuous improvement of nutrition programs to address micronutrient deficiencies in rural communities.

## Introduction

Food insecurity leading to malnutrition is a growing problem for individuals and communities which lack consistent access to a sufficient quantity and quality of food, with this issue gaining attention from donors and governments, particularly in low -and middle-income countries (LMICs) [1]. The underlying causes of malnutrition may include lack of community awareness, poor literacy, lack of resources, inadequate availability of vegetables and fruit, and resulting poor nutritional practices, and low purchasing power in households, which in turn negatively affects overall health in LMICs such as Pakistan [2]. Food insecurity and poor nutrition primarily impact vulnerable groups—especially women and children in impoverished areas—leading to pregnancy complications, anemia-related work limitations, compromised growth and cognition, and heightened health risks for mothers and their offspring [3,4].

Pakistan’s economy is agricultural based and more than 65% of the Pakistan’s population depends on agriculture for its livelihood [5]. According to the Cost of Basic Needs (CBN) approach, which focuses on the consumption patterns of households, about 24% of Pakistanis live below the poverty line, not being able to afford sufficient food to meet the recommended minimum benchmark diet of 2350 Kcals per day [6]. Balochistan is the poorest of Pakistan’s provinces with the worst health indicators in the country and 52% of the population living below the poverty line. Health and nutrition are major problems in the province which has the highest prevalence of malnutrition with stunting rates as high as 47% in children under the age of 5 years [7]. Many districts in Balochistan are food insecure, with women and children being most vulnerable to food insecurity and malnutrition. There are multiple determinants of stunting and malnutrition across districts in Balochistan that pose challenges to improving nutrition, particularly for vulnerable populations. This includes the geographical isolation of districts from the capital, the relative isolation of houses within districts, limited crop production, lack of water, lack of basic health facilities, low literacy and limited exposure to information and the outside world. Improving food and nutritional security and quality of diets in food insecure populations requires effective and efficient strategies that efficiently use available resources and have long term benefits [8,9].

Understanding how deeply connected the food and agriculture sector is to underlying causes of malnutrition, various agricultural tools can be used to improve the health and nutrition of people afflicted with malnutrition. Fortification is one tool that is currently being employed to address malnutrition among resource poor families in the developing world. However, poor technology, lack of economic capacity and lack of regulations and food laws are some cited barriers that are not conducive in creating an enabling environment for fortification [10]., Thus, these strategies may not be acceptable or sustainable in remote low socioeconomic communities even where the burden of nutritional deficiencies is immense. Likewise providing supplementation in large scale nutrition programs could be challenging especially if the food is to be taken regularly over a long time period and is limited by a dependency on international funding, an inability to reach most vulnerable populations, unreliable and inconsistent delivery systems, and dependence on individual compliance [11]. Additionally, nutrient supplementation should not be considered as a substitute to a well-balanced diet, with fortification, also limited by local purchasing power and distribution systems [12].

There is increasing recognition that the problem of malnutrition is multi-faceted and requires actions with a greater focus on poverty alleviation, improvement of mother’s health literacy, including community-based education and targeted nutritional interventions. Emerging evidence from South Asia and elsewhere demonstrates a potential for nutrition sensitive agricultural interventions to improve nutritional outcomes [13]. Kitchen garden programs in particular have shown to be significant intervention in improving food security, nutritional status and household income [14,15]. Kitchen garden program’s introduction of livestock and backyard poultry have also been associated with improved dietary diversity scores, greater consumption of vitamin A rich fruits/vegetables/pulses, and other fruit and vegetables, as well as improved complementary food availability [16,17]. Studies identify the need for interventions to be context-specific, be able to stimulate ideas to incorporate nutritional education parameters, and family farming system approaches to ensure sustainable impacts [11,18].

The Food and Agriculture Organization (FAO) of the United Nations, together with the Department of Agriculture and Cooperatives of the Government of Balochistan piloted an “Integrated Kitchen Gardens” program in Balochistan province in 2015. The project’s primary aim is to provide opportunities to low income rural community women to engage in economic activities and eventually improve food security and nutrition for their families. A secondary objective of the program is to increase the nutritional status of women and children under the age of five years through kitchen garden and nutrition education. FAO has established vegetable kitchen gardens in 6 districts in South West Balochistan.: Chaghai, Kech, Kharan, Nushki, Panjgur, and Washuk. Adopting a Farmer Field School (FFS) approach on the establishment and operation of these gardens FAO equips women with knowledge and skills to maintain their own gardens back home in their districts. Kitchen garden interventions have been shown to be instrumental in increasing rural production and consumption of vegetables and fruits and to enhance the nutritional status of mothers and children in the rural villages in many countries [19,20]. However, there is currently little evidence to identify if nutrition-sensitive agricultural interventions in Pakistan can significantly impact on nutrition and health outcomes.

As the global community and national governments seek to end hunger and poverty under the Sustainable Development Goals Agenda 1&2, it is important to gain an improved understanding of whether and how nutrition sensitive agriculture interventions can generate nutritional impacts in food insecure areas [21]. Thus, the aim of this study was to better understand if nutrition sensitive interventions of kitchen gardens would be effective in the Balochistan cultural context to change patterns of food consumption and responsiveness to dietary diversity, agro-biodiversity, and nutrition and health, addressed within the program. Specific objectives of the study were to compare nutrition and health AKAB of women who attended the kitchen garden program trainings and interventions against a “control group” of women who did not attend the kitchen garden program trainings and were unaware of the interventions.

## Materials and methods

### Study setting, population, and design

The present study was conducted among the rural Pakistani women with children under five years of age, to assess their AKAB towards nutrition and health. The study was conducted between April-August 2019 following the completion of the integrated kitchen garden training and interventions. The majority of the population in Balochistan is located in rural areas and principal livelihood activities include agriculture and livestock production. Households in remote areas are spaced well apart with the province experiencing arid weather patterns and a semi-arid land with mostly dry climate, while the soils are still conducive to cultivation [20].

A community based cross-sectional survey (*N* = 209) was conducted among participants in the study area using a two-stage cluster sampling method. The study used two stage cluster sampling technique. In the first stage, segmentation of the two district villages (Nushki and Kharan) from 6 FAO intervention districts in South-West Balochistan namely, Chaghai, Kech, Kharan, Nushki, Panjgur, and Washuk was done. In the second stage, sample selection involved randomly assigning twenty villages in the two districts from 10 Union Councils in Nushki district, and 7 Union Councils in Kharan district, being selected as the study sites. Union councils are the smallest administrative units in Pakistan. Within the randomly selected villages from each district, every fourth household was targeted until the required sample was attained from each district. In cases where participants were not available within the selected household, the adjacent household was visited. One female from each of the households was selected for the face-to-face survey.

The key indicator used to calculate the sample size was the “expected proportion of awareness of kitchen gardens”. Based on a previous quantitative knowledge, attitudes, and practice (KAP) survey conducted in Pakistan [22] using the power calculation alpha = 0.05 (1- β = 0.90). With a medium size effect, the minimum required total sample size was 164 participants. To allow for the expected attribution rate inefficiency, the sample was upsized to 200 households in both districts. A total of 225 participants were approached from the two districts with 209 women included in the final analysis after excluding incomplete responses (response rate: 92.8%). The 209 participants were then divided into two groups: An “intervention group” (n = 114) of women who attended the kitchen garden program; and a “control group” (n = 95) of women in the same districts who did not attend the kitchen garden program and did not recall FAO interventions. For this study the “control” group was those women from the two study districts who had not participated or enrolled by FAO in the kitchen garden program/campaign and received no training in FFS. This was confirmed through the list maintained by FAO office in each of the six districts where the kitchen gardening program was initiated.

### Kitchen garden project

The Kitchen Gardens Program was funded by AusAID and the FAO and ran from 2015–2020 [23]. Initial aspects of the program included social and behaviour change communication (SBCC) formative research with program beneficiaries to support the kitchen garden training and other program interventions [24]. This included the pre-testing, development and dissemination of community resources including flip charts, food mats, posters, and mobile truck art as well as development and pretesting of the preferred branding for the program–“My Garden My life”. Preferred message channels identified from the formative research included interpersonal communication (IPC), through opinion leaders such as doctors and lady health workers, WhatsApp groups, and the use of other support groups, with mass media channels such as radio and television also recommended channels of communication to promote kitchen garden nutrition messages.

The kitchen garden training component focused on training women and some men farmers. A total of 350 community kitchen gardens were established in six districts in South West Balochistan including Chagai, Kech, Kharan, Nushki, Panjgur, and Washuk districts. Women from these districts were recruited to the Farmer Field Schools (FFS) to learn new gardening concepts for the establishment of community kitchen garden. This included experimenting with different planting times, seed varieties, reticulation systems, fertilizer, pest management, solar drying incubators, and planting techniques. Supporting interventions included strengthening of value chains and livelihoods systems and connecting farmers with markets for collective marketing of commodities such as onions, goats, sheep, poultry, wool and dates and to enhance the overall enabling environment. The kitchen garden program evaluation commenced within 4 weeks following completion of the final trainings.

### Measures

The survey questionnaire assessed: (i) Socio-demographic background of participants and household information; (ii) Awareness was a key initial measure, based on participant recall of nutrition and health messages from community-based campaign materials/announcements, trainings or interpersonal communication (IPC) messages on nutrition and health delivered through health/community workers or other influencers; (iii) Knowledge, attitudes and behavioural intentions/behaviours towards nutrition and health; and (iv) Sources of information and preferred communication channels for nutrition health literacy. The questionnaire development process included insights from a literature review, formative research inputs, multi-stakeholder meetings with the project implementers, and piloting the questionnaire to identify any potential issues or areas for improvement before its finalization for data collection. Cronbach’s alpha coefficients were used to assess the questionnaire’s reliability and internal consistency for attitudes and behavioral dimensions measured through 5- point Likert scales while questions on knowledge constructs were measured through dichotomous binary scales. Cronbach’s alpha coefficient values ranging from 0 to 1 and a scoring above 0.7 are generally regarded as acceptable. The questionnaire was developed in English and then translated into Urdu. Following pre-testing with local respondents, the Urdu version of the questionnaire was revised and back translated into English to assure equivalence of items and scales was maintained [25].

*‘Awareness’*, regarding nutrition and health was assessed by women’s ability to correctly recall messages unprompted and also following prompting of the range of SBCC interventions. All participants were asked to recall any announcements or messages from community-based campaign materials/announcements, trainings, IPC messages on nutrition and health delivered through health/community workers or other influencers. Firstly, participants were asked to spontaneously recall and describe any community-based communication campaigns messages received in the previous six months. All those who recalled any nutrition and health message were recorded as “unprompted recall”. Next, all respondents were shown images from the trainings and messages disseminated through community and IPC channels and were asked if they recalled the messages. All those who recalled any messages from the prompt cards and in addition were able to recall message components were defined as having “prompted recall”. Respondents identified as being aware either through unprompted or prompted recall were designated as Program ^Aware^ vs. those who did not recall any message were designated as Program ^Unaware^_._

*‘Knowledge’* indicators contained seven dichotomous items on good diet and nutrition, the risks of malnutrition for women and children, causes and prevention of iron deficiency anemia (IDA). The Cronbach’s alpha score for the knowledge domain (0.712) reflected an adequate internal consistency.

*‘Attitudes*’ towards nutrition and health, and kitchen garden programs included 5-point Likert scales examining personal and child risk to malnutrition, attitudes toward food diversity and developing their own kitchen garden, attending the kitchen garden trainings, introducing healthy food options and vegetables and attitudes toward improving food security. *‘Behavioural intentions’* towards nutrition and health were also measured using 5-point Likert scales. Reported behavioural intentions were assessed using: 1) dietary diversity (2) seeking information and advice for nutrition issues and kitchen gardening. The Cronbach’s alpha coefficient for the attitude domain was 0.936 and behavioral domain was 0.823 respectively. With these domains demonstrating good internal consistency. *‘Socio-demographics’* included information on participant age and number of children, educational status, monthly family income, home status, available water sources, decision making authority over food in the household, and preferred sources of health information.

### Data collection

The quantitative survey questionnaire was administered by 14 trained enumerators under the guidance of 2 research assistants supervised by the study researchers. The survey team was identified from members of local communities, familiar with the area and local languages. Prior to data collection, a one-day training session was conducted which included briefing on the project and its objectives. The training ensured that fieldworkers understood the protocols for employing and recording item probes and that all items were clearly understood. Finally, mock interviews and pilot testing of the survey instrument were conducted, and adjustments made prior to going into the field. All participants were required to give their verbal consent prior to completing the survey.

### Statistical analysis

Demographic and baseline characteristics were summarized using frequency and percentages for categorical variables and means and standard deviations for quantitative variables. Socio-demographic characteristics and key outcome variables of participants were compared between the two groups (intervention group vs control group) using paired t-test for continuous variables and Chi-square test for categorical variables. Results were reported as p-values and significant criteria were set at 95%*, 99%** and 99.9%*** confidence levels. All analyses were carried out using software SPSS version 21.

### Ethics approval

Strict ethical standards were maintained during the design, fieldwork implementation and analyses of the study design in line with recommended approaches for studies conducted with vulnerable populations in LMIC settings [26]. Approval from Dow University of Health Sciences Institutional Review Board (IRB) was obtained prior to conducting the study (IRB-/399/DUHS/Approval/2019).

## Results

### Socio-demographic characteristics

A comparison of socio-demographic characteristics between women attending the kitchen garden program (n = 114) and women not attending kitchen garden program (n = 95) identified differences between groups (Table 1). Out of a total, 62.2% (n = 130) of households contained a woman from 18–35 years age; with a mean age of 33.4 years (SD ±7.8 years). The average family size was 8 members, with a standard deviation of ± 3.39. Most women had no formal education (60.8%, n = 127) and 92.8% (n = 194) were living in a house made of mud walls (‘Kacha house’). About half of the women (46.9%, n = 98) reported tube well/bore hole as the main source of drinking water. Electronic media (TV/Radio) (26.8%, n = 56) and mobile phones (12.9%, n = 27) were identified as the main sources of information in households. Approximately 32.5% (n = 68) women were the household decision makers for health and food preparation. Significant differences (p<0.05) were observed for access to print media (4.8%, n = 10), and social media (6.7%, n = 14) with mostly women being the beneficiaries of the kitchen garden program.

**Table 1:** Socio-demographic characteristics of women attending Kitchen Garden and those not attending Kitchen Garden programs and interventions (N = 209).

Characteristics		Total	Women attending Kitchen Garden	Women not attending Kitchen Garden	
					*P*- value*
		(*N* = 209)	(*n* = 114)	(*n* = 95)	
		n (%)	n (%)	n (%)	
**Age category**					
	18–35	140 (67)	81 (71)	59 (62.1)	0.510
	36–45	59 (28.2)	27 (23.7)	32 (33.7)	
	46–55	7 (3.3)	4 (3.5)	3 (3.2)	
	56 or greater	3 (1.4)	2 (1.8)	1 (1.1)	
**Education level**					
	No formal schooling	127 (60.8)	70 (61.4)	57 (60.0)	0.729
	Madrassa (Religious school)	43 (20.6)	25 (21.9)	18 (18.9)	
	Primary school	20 (9.6)	8 (7.0)	12 (12.6)	
	Secondary/high school	12 (5.7)	7 (6.1)	5 (5.3)	
	College/University	7 (3.3)	4 (3.5)	3 (3.2)	
**Monthly household income (PRs)**					
	10,000 or less	128 (61.2)	70 (61.4)	58 (61.1)	0.889
	10,001–20,000	47 (22.5)	24 (21.1)	23 (24.2)	
	20,001–30,000	11 (5.3)	7 (6.1)	4 (4.2)	
	30,001 or greater	23 (11.0)	13 (11.4)	10 (10.5)	
**Home status**					
	Kacha (Mud home)	194 (92.8)	106 (54.6)	88 (45.4)	0.922
	Pakka (Brick made home)	15 (7.2)	8 (53.3)	7 (46.7)	
**Source of drinking water**					
	Household connection	31 (14.8)	16 (51.6)	15 (48.4)	0.586
	Tube well or borehole	98 (46.9)	49 (50.0)	49 (50.0)	
	Protected spring	11 (5.3)	8 (72.7)	3 (27.3)	
	Bottled water or filter	3 (1.4)	2 (66.7)	1 (33.3)	
	Public tap or stand pipe	58 (27.8)	34 (58.6)	24 (41.4)	
	Protected dug well	6 (2.9)	3 (50.0)	3 (50.0)	
	Rainwater	2 (1.0)	2 (100)	-	
**Decision maker for health/food**					
	Self (Wife)	68 (32.5)	38 (55.9)	30 (44.1)	0.262
	Husband	64 (30.6)	35 (54.7)	29 (45.3)	
	Mother-in-law	24 (11.5)	11 (45.8)	13 (54.2)	
	Father-in-law	33 (15.8)	15 (45.5)	18 (54.5)	
	Any one of them	20 (9.6)	15 (75.0)	5 (25.0)	
**Main sources of information**					
	Electronic media (TV/Radio)	56 (26.8)	23 (41.1)	33 (58.9)	0.021
	Mobile phone	27 (12.9)	20 (74.1)	7 (25.9)	
	Print media (Newspaper/Magazines)	10 (4.8)	8 (80.0)	2 (20.0)	
	Both (Electronic media & mobile phone)	102 (48.8)	54 (52.9)	48 (47.1)	
	Social media (Whatsapp/FB)/others)	14 (6.7)	9 (64.3)	5 (35.7)	
**Communication channel preferences**					
	Print media	15 (7.1)	14 (12.3)	1 (1.1)	<0.001
	Electronic media	29 (13.8)	28 (24.6)	1 (1.1)	
	Social media	69 (33.0)	34 (29.8)	35 (36.8)	
	Social referents	92 (44.0)	57 (50.0)	35 (36.8)	
	Community referents	18 (8.6)	15 (13.2)	3 (3.2)	
	Health workers	44 (21.0)	26 (22.8)	18 (18.9)	
	NGOs	138 (66.0)	89 (78.1)	49 (51.6)	
**Total family members (mean ± SD)**		7.95 ± 3.39	7.56 ± 3.66	8.42 ± 2.97	0.068

*P-value calculated using Chi-squared analysis and two means comparison analysis (t-test).

Respondents’ preferences on the choice of preferred communication channels for nutrition and health related messages they would receive were also examined in order to provide insights into how health messages could be more effectively targeted to vulnerable populations. The most preferred channels of communication on nutrition and health were through IPC channels including NGO staff (66%, n = 88); particularly FAO field workers, and other social referents (44%, n = 92), while Social media was also preferred by 33% (n = 69) (see Table 1).

### Awareness and knowledge

The results (represented in Table 2) identified that women attending kitchen garden were more Program^aware^ compared to those who did not attend the kitchen garden program trainings (65.8%, n = 75 vs 36.8%, n = 35; *p*<0.001). Similarly results on knowledge indicators also identified significant differences between women attending kitchen garden and women not attending kitchen garden, with attendees more knowledgeable about the lack of nutrient rich foods and nutrient deficiencies causing: serious illness (81.6%, n = 93; p<0.001), Iron deficiency/Anemia in mother and children (86.0%, n = 98; p<0.001), and pregnant women and unborn child health complications (85.1%, n = 97; p<0.001) with 69.3% of respondents with kitchen garden replied correctly that delivery complications are caused by being undernourished (n = 79, p = 0.030). However, there was a gap in participants’ knowledge regarding the mental health consequences and maternal and infant mortality due to poor nutrition.

**Table 2:** Nutrition program awareness and knowledge regarding nutrition and health risks among women attending Kitchen Garden and those not attending Kitchen Garden.

Awareness of health and nutrition interventions		Total		Women attending Kitchen Garden		Women not attending Kitchen Garden		
		n	%	n	%	n	%	*P*-value
	Program^Aware^	110	56.2	75	65.8	35	36.8	<0.001
	Program ^Unaware^	99	47.4	39	34.2	60	63.2	
**Nutrition and health knowledge: Lack of proper food and nutrition can cause the following:**								
	Serious illnesses	147	70.3	93	81.6	54	56.8	<0.001
	Physical growth of children (underweight & stunted)	159	76.1	90	78.9	69	72.6	0.287
	Mental disability in children	127	60.8	67	58.8	60	63.2	0.518
	Pregnant women & unborn child health complications	156	74.6	97	85.1	59	62.1	<0.001
	Delivery complications	131	62.7	79	69.3	52	54.7	0.03
	Iron deficiency/Anemia in mother and child	159	76.1	98	86	61	64.2	<0.001
** **	Maternal and child mortality	105	50.2	63	55.3	42	44.2	0.112

P-value calculated using Chi-squared test.

### Attitudes, and behavioural intentions

Descriptive statistics were conducted on five-point Likert scales to identify attitudes, behavioural intentions and reported behaviours of women attending kitchen garden, and those not attending kitchen garden. Findings identified significant differences with women attending kitchen garden having more positive attitudes across all items including intentions to grow food in their kitchen garden (p = 0.001), improve dietary diversity (p<0.001), reduce the amount of meat in the family diet (p<0.001), learn more about improving food and nutrition security (p<0.001), and the health impact of food and nutrition (p<0.001). Additionally, significant differences were identified with kitchen garden participants over those not attending kitchen garden on attitudinal indicators (p<0.001), with high mean scores identified across all indicators by intervention group participants (4.03±0.74–4.62±0.74). Findings for behavioural indicators identified significant differences between kitchen garden intervention and control groups with regard to items: We eat vegetables every day (*p*<0.001), I eat a variety of vegetables every day (*p* = 0.003), and health seeking behaviours: If I have any questions about food, nutrition and health issues, I’m able to get information and advice from local sources (health workers, local leaders community members (*p*<0.001), while one item on fruit consumption identified no significant differences (*p* = 0.195). (Table 3).

**Table 3:** Attitudes, behavioral intentions and nutrition and health behaviours of female participants attending Kitchen Garden and those not attending Kitchen Garden.

Attitudes	Total	Women attending Kitchen Garden	Women not attending Kitchen Garden	
	Mean ± SD	Mean ± SD	Mean ± SD	P-value
I am planning to set up my own kitchen garden for good nutrition and health	4.10 ± 1.20	4.51 ± 0.89	3.62 ± 1.34	0.001
I am planning to attend kitchen garden training	3.97 ± 1.19	4.35 ± 0.98	3.51 ± 1.26	<0.001
I am planning to introduce more vegetables and fruits in diet	3.90 ± 1.16	4.32 ± 0.81	3.39 ± 1.30	<0.001
I am planning to reduce meat intake of my family	3.69 ± 1.21	4.03 ± 0.96	3.29 ± 1.34	<0.001
I am planning to learn more about improving food & nutrition security	3.99 ± 1.24	4.47 ± 0.88	3.41 ± 1.35	<0.001
I want to learn more about health impact of food & nutrition	4.03 ± 1.26	4.62 ± 0.74	3.36 ± 1.41	<0.001
**Behavioural intentions/Behaviours**	**Total**	**Mean ± SD**	**Mean ± SD**	**P-value**
Homegrown Vegetables taste fresher than bought from a market	3.22 ± 1.63	4.22 ± 1.09	2.02 ± 1.33	<0.001
Homegrown Vegetables are cheaper than bought from market	3.28 ± 1.63	4.31 ± 0.99	2.05 ± 1.37	<0.001
We eat fruits every day	3.69 ± 1.22	3.79 ± 1.24	3.57 ± 1.20	0.195
We eat vegetables every day	3.84 ± 1.25	4.22 ± 0.96	3.39 ± 1.40	<0.001
I eat a variety of vegetables (e.g., lettuce, cabbage, tomatoes, carrots, etc.), every day	4.30 ± 1.15	4.52 ± 0.88	4.04 ± 1.36	0.003
If I have any questions about food, nutrition and health issues, I’m able to get information and advice from local sources (health workers, local leaders community members)	3.85 ± 1.21	4.17 ± 1.02	3.46 ± 1.32	<0.001

Scale 1: Disagree completely; 2: Somewhat disagree; 3:Neither agree nor disagree; 4: Somewhat agree; 5: Completely Agree.

*P*-value calculated using Independent t-test.

### Association of Kitchen Garden interventions with participant AKAB

Lastly, the role of kitchen garden interventions on AKAB was examined using univariate and multivariate regression analyses. Univariate analysis (model 0) indicated that women who attended kitchen garden were more likely to be Program^aware^ (OR = 2.54, 95%CI 1.07–6.00, *p* = 0.033) compared to women who did not attend the kitchen garden program. The association remained significant following adjusting for sources of information, the only significant explanatory variable in the model1 (OR = 3.92, 95%CI 1.13–13.51, *p* = 0.031). Similarly, univariate analysis (model 0) identified that women attending kitchen garden were more likely to have higher knowledge, positive attitudes, behavioural intentions and towards nutrition and health (OR = 1.30, 95%CI 1.12–1.51, *p*<0.001), (OR = 4.55, 95%CI 2.97–6.99, *p*<0.001) and (OR = 2.99, 95%CI 2.05–4.37, *p*<0.001) respectively. The association remained significant in model 1(OR = 1.59, 95%CI 1.27–1.99, *p*<0.001), (OR = 4.86, 95%CI 2.77–8.53, *p*<0.001) and (OR = 1.98, 95%CI 1.26–3.12, *p* = 0.003) for knowledge, attitudes, and behavioural intentions/behaviours respectively (Table 4).

**Table 4:** Univariate and Multivariate analysis of Kitchen Garden intervention with AKAB of female participants.

		Model 0			Model 1		
		β (S.E)	cOR (95% CI)	*p*-value	β (S.E)	aOR (95% CI)	*p*-value
**Awareness**							
	Program ^Unaware^	Ref.			Ref.		
	Program^Aware^	0.93 (0.43)	2.54 (1.07–6.00)	0.033	1.36 (0.63)	3.92 (1.13–13.51)	0.031
**Knowledge**		0.26 (0.07)	1.30 (1.12–1.51)	<0.001	0.46 (0.11)	1.59 (1.27–1.99)	<0.001
**Attitudes**		1.51 (0.21)	4.55 (2.97–6.99)	<0.001	1.58 (0.28)	4.86 (2.77–8.53)	<0.001
							
**Behavioural intentions/Behaviours**		1.09 (0.19)	2.99 (2.05–4.37)	<0.001	0.68 (0.23)	1.98 (1.26–3.12)	0.003

Model 0: Unadjusted models signify the bivariate association between dependent and independent variables alone.

Model 1: Adjusted analysis is a reduces model taking into account the only covariate that showed a significant association on univariate analysis; SE: Standard error; cOR: crude odds ratio; aOR: Adjusted odds ratio; CI: Confidence intervals.

## Discussion

This study provides important insights into the impact of the kitchen garden program on the AKAB of women in rural Balochistan. Recall of and knowledge about nutrition and health were found to be significantly greater among women who attended the kitchen garden program versus those who did not. The findings also identified that households already engaged in kitchen gardening are also more likely to produce and consume nutrient rich foods and have more knowledge about nutrition and health within the households. Similar findings were also observed in rural India and Tanzania where successful implementation of kitchen garden programs and trainings demonstrated greater knowledge among householders attending the interventions [27,28]. Hence, nutrition education can be an important tool to improve dietary diversity in vulnerable groups [29].

Significant differences in awareness towards nutrition and health aspects were found in women attending kitchen garden compared to women not attending kitchen garden. Moreover, the kitchen garden interventions were more profound for critical behavioural determinants such as behavioural intentions and reported behaviours toward growing their own food and attending trainings on gardening. This emphasizes the potential for improved attitudes, competencies, and skills toward establishing kitchen gardens by vulnerable women who recalled the kitchen garden program interventions. Further, it was found that the majority of both the intervention and control groups were in agreement with regard to the importance of growing their own food and had a stronger preference for IPC channels of communication for the acquisition of knowledge and skills on developing kitchen garden and improving nutrition and health outcomes. Also the findings from this study confirm the need for nutrition specific approaches that address the immediate causes of malnutrition [30,31]. This SBCC intervention approach which combined a range of IPC, training, incentives and community and mass media supports has demonstrated that improvements in the confidence of and competencies of vulnerable groups can be achieved within relatively short time periods.

Another observation was that households attending kitchen garden consumption of vegetables and fruits also increased. They consumed more green leafy, vitamin A rich vegetables, compared to the control group with the same socioeconomic backgrounds, living in the same districts. Moreover, consumption of vegetable and fruit was found to be higher than meat intake among the majority of the women attending kitchen garden. Their intake supported daily recommendations of 2 fruits and 5 vegetables [32]. The positive link between kitchen garden and improved dietary diversity is in line with findings from similar interventions with vulnerable populations in Africa [33]. In terms of the local context data from Pakistan suggests that households attending kitchen gardening have greater intake of home-produced nutrient rich vegetables and fruits, high nutrition literacy, access to inexpensive and fresh vegetables supply during summer and winter seasons [34,35]. This study builds on the existing literature by using a novel approach to identification of intervention and control groups for the kitchen garden sample frames. This involved the identification of program^aware^ and program^unaware^ participant segments which were approaches applied to a TB advocacy, communication and social mobilization (ACSM) post intervention study also conducted in Pakistan [22]. Thus, our study has demonstrated the efficacy of adapting the study methodology in Balochistan context given the considerable challenges in implementing RCTs in such environments.

The application of SBCC impact assessment AKAB indicators, combined with nutritional data collection, provides a broader range of program performance indicators for continuous improvement of kitchen garden interventions including learnings on how different audience segments may respond to the program activities and their preferred channels of nutrition and health communication. The SBCC approaches also identified where participants reside on behaviour change continuum, identifying measures of cognition (awareness) of the health problem, knowledge, motivation, skills and competencies which may ultimately lead to maintenance and advocacy for the kitchen garden program’s desired behavioural objectives. Findings demonstrate that improvements in all key indicators are possible within a relatively short time period of 3 months of the pilot program. Lessons learned include the need to further refine indicators and evaluation methodologies to account for the resource constrained settings in which the programs are implemented and the need to institutionalize monitoring, evaluation, learning and adaptation (MELA) as a core component in strategies to address malnutrition and food insecurity.

Future study approaches could focus more on critical issues in identifying what has been termed the “dose/response relationship” of SBCC. This may lead to a better understanding of the impact of SBCC on participants who may have had only one level of message exposure compared to those who may have recalled multiple messages and interventions, in order to identify the optimal number of contacts needed in order to attain significant impact on the targeted behaviors [30]. Additionally, the scaling-up of innovative SBCC approaches incorporating kitchen garden trainings and incentives were identified as well as the need to develop improved impact assessments for SBCC strategies and opportunities for national and regional efficiencies in adapting existing, best practice SBCC creative and programming approaches [36].

Limitations of our study included resource constraints, geographic isolation, insecurity, and distances to households. These limitations restricted the duration of the fieldwork and sample sizes to a smaller number of households, which restricted case numbers for some statistical tests and additional segmentation analyses. Limitations are also acknowledged in the case/control approach given true randomization of the intervention/experimental and control group was not feasible in the resource constrained and insecure settings, coupled with the potential for control group participants being exposed to and recalling mass media SBCC components. However, the study approach adopted shows promise for similar post-intervention surveys of nutrition sensitive interventions implemented in challenging environments. Other possible limitations include selection bias in unobservable characteristics, and social-desirability bias in the face to face interviews, as women may have over reported their consumption of healthy food and underreported less healthy foods. However, the enumerators were trained to carefully explain the study purpose prior to data collection, and participants assured about confidentiality and anonymity, which may have reduced the potential of biases.

It is also important to consider the possible influence of other health nutrition campaigns that were going concurrently in the community. Nonetheless the participants exposed to FAO, kitchen garden trainings and campaign messages had higher scores on measures of concern towards health and nutrition, suggesting that there is a real effect of the kitchen garden program. As future kitchen garden campaign rolls out on a larger scale, a fuller examination of their impact on awareness, knowledge, and additional elements such as attitudes, and behaviors are required, using longitudinal study designs. Last, it is possible that participants who were more concerned about nutrition were also more attentive to the issue, and thus more likely to correctly recall the SBCC messages, following prompting.

## Conclusions

The evaluation of the kitchen garden pilot program holds great promise for supporting nutrition and health improvements and uplifting the scale of nutrition sensitive interventions. The findings from the kitchen garden pilot program have demonstrated the potential to achieve measurable behavioral impact through the utilization of behaviours centered approaches, while acknowledging the behavioural determinants which may impact on change within complex cultural environments. Additionally, the study method provides a novel, resource efficient and academically rigorous approach to the evaluation of evidence based, agro-biodiversity interventions to reduce food insecurity, an issue which will require greater investment in coming years. Given that malnutrition and food insecurity are global challenges that are affecting a growing number of communities across the world, the evaluation findings can contribute to collective efforts in addressing these challenges by offering evidence on the effectiveness of specific strategies like kitchen garden programs supported by nutrition sensitive SBCC interventions.

It is acknowledged that kitchen garden interventions require considerable resources such as time and funding to build trust and engagement toward establishing gardens, particularly in conservative and isolated rural communities like those encountered with this pilot program. Further evaluation studies, including pre- and post-intervention designs could be considered to better detect changes over time, and required adaptation as the program evolves. Findings may support the government, the FAO and other stakeholders in Balochistan to further develop evidence-based, longitudinal studies to assess the impact of nutrition sensitive intervention programs and policies. The impact evaluation of a kitchen garden program in rural Pakistan has the potential to contribute valuable insights to the broader global discourse on nutrition, food security, and sustainable agricultural practices. Its findings can inform decision-making, inspire innovations, and foster collaboration among stakeholders worldwide to address common challenges. Recommendations for further program improvement include important questions of sustainability, scale-up, program barriers and opportunities, and return on investment with kitchen garden program expansion.

## Supporting information

S1 Data (XLSX)
